# Bioaccessibility of Cafestol from Coffee Brew: A Metabolic Study Employing an *In Vitro* Digestion Model and LC-HRMS

**DOI:** 10.1021/acs.jafc.4c06411

**Published:** 2024-12-04

**Authors:** Ana Brand, Ana Silva, Cyrus Andriolo, Caroline Mellinger, Thaís Uekane, Rafael Garrett, Claudia Rezende

**Affiliations:** a Instituto de Química, Universidade Federal do Rio de Janeiro, Rio de Janeiro 21941-909, Brasil; b L’Oréal Brazil, Rio de Janeiro 21044-020, Brasil; c EMBRAPA Agroindustria de Alimentos, Rio de Janeiro 23020-470, Brasil; d Departamento de Bromatologia, Escola de Farmácia, Universidade Federal Fluminense, Niterói, Rio de Janeiro 24241-002, Brasil

**Keywords:** coffee, diterpenes, bioaccessibility, static *in vitro* digestion, metabolism, high-resolution mass spectrometry

## Abstract

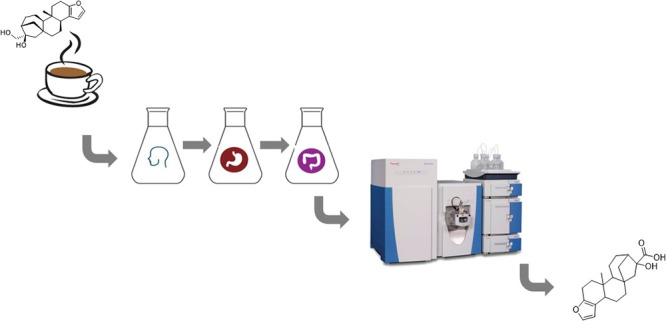

Cafestol is an *ent*-kaurene skeleton diterpene that is present in coffee beans and brews. Although several biological activities have been described in the literature for cafestol, such as hypercholesterolemic, anti-inflammatory, anticerous, and antidiabetic effects, its metabolism within the human body remains poorly understood. Therefore, this study aimed to quantify cafestol in boiled coffee brew, assess its bioaccessibility using a static *in vitro* digestion model, and investigate the metabolites formed during the digestion process using liquid chromatography coupled to high-resolution mass spectrometry. Cafestol content in the boiled coffee brew ranged from 127.47 to 132.65 mg L^–1^. The bioaccessibility of cafestol from boiled coffee brew using the *in vitro* digestion model was 93.65%; additionally, in the intestinal phase, cafestol was mainly found in its alcohol form. Additionally, a novel carboxylic acid derivative metabolite from cafestol with *m*/*z* 331.1909 [M + H]^+^ formed in the oral digestion phase is proposed. This metabolite was also detected in other digestion phases. Thus, this is the first article to investigate the metabolism of cafestol during digestion using an *in vitro* digestion model. The results indicate that cafestol is bioaccessible, is available to absorption, in its alcohol form, and suffers an oxidation reaction during the oral phase of digestion.

## 1 Introduction

Coffee brew is one of the most consumed beverages in the world due to its desirable flavor and aroma.^1^ According to the International Coffee Organization, 170.3 million 60 kg bags of coffee were consumed in 2022 (ICO, 2024). Brewed coffee is prepared using roasted coffee beans and hot water and is usually consumed as a hot beverage, although cold variations such as cold brewed coffee are becoming more popular.^2^ Coffee brew is rich in bioactive compounds such as caffeine, chlorogenic acids, trigonelline, and diterpenes.^3,4^

**Figure 1:**
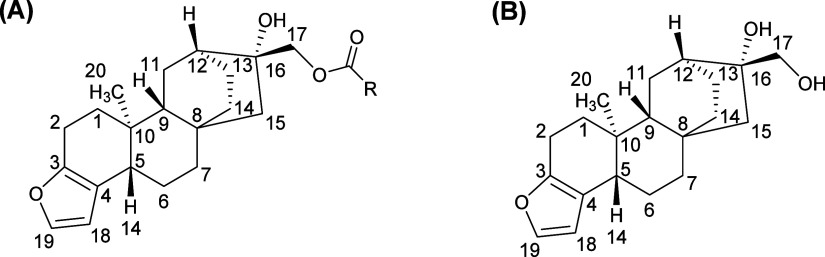
Chemical structure of (A) cafestol ester—R corresponds, mainly, to palmitoyl and linoleoyl moieties—and (B) cafestol alcohol (free).

Cafestol is an *ent*-kaurene diterpene found in the lipid fraction of coffee beans.^5^ The majority of cafestol is esterified with acyl moieties, mainly palmitoyl and linoleoyl units; only a small fraction occurs as free diterpene alcohols (Figure 1).^6^ Total cafestol content in coffee brews varies greatly according to the brewing method because of its low solubility in water and the use (or not) of a filter to prepare the beverages.^7,8^ Boiled coffee brews have the highest cafestol content (692 mg L^–1^), while filtered coffee brews have the lowest (3.5 mg L^–1^).^9^ The boiled coffee brew is an adaptation of the Turkish coffee preparation; it consists of boiling coarsely ground roasted coffee beans in water for 10 min and waiting 2–5 min before consumption. This type of coffee brew is mostly consumed in Scandinavian countries.^8^

Cafestol exhibits diverse beneficial biological activities, including anti-inflammatory, antioxidant, antidiabetic, and anticarcinogenic effects. However, it also has a pronounced hypercholesterolemic effect.^3,10^

Despite the popularity of coffee brews and the many biological activities described for cafestol, a few studies have been conducted on the metabolism of this diterpene in humans. The first study describing the pharmacokinetics of cafestol was published in 1998.^11^ The authors studied the absorption and excretion of cafestol after the ingestion of a boiled coffee brew by ileostomy volunteers. Upon analysis of the contents of the ileostomy bags, the researchers determined that 67% of administered cafestol was absorbed into the duodenum. Additionally, 24% was lost in the stomach, and only 1.2% was excreted in the urine as sulfate or glucuronide conjugates 8 h after ingestion. While the authors observed a decrease in the amount of cafestol during the digestion process, they did not further investigate the metabolites formed. Another study from 2019, investigated the bioaccessibility of cafestol from spent coffee grounds using and an *in vitro* digestion model.^12^ The authors found that cafestol has a low bioaccessibility of 13.39%. This is the only work in the literature investigating cafestol metabolism employing an *in vitro* digestion model. Other studies focused on the biotransformation and distribution of cafestol and explored the metabolism of cafestol in mice through intravenous administration of cafestol through the portal vein; bile excretion aliquots were collected and analyzed. The researchers identified a possible cafestol metabolite identified as a hydroxy-cafestol conjugated to glutathione.^13^ Subsequently, using the same methodology, the authors investigated the absorption and distribution of deuterated [3H]-cafestol via autoradiographic analysis in mice.^14^ They found that deuterated [3H]-cafestol was absorbed into the duodenum. This study also highlighted the liver as a site of cafestol metabolism with subsequent elimination through bile. Furthermore, four cafestol metabolite candidates were observed in bile; however, the only identified metabolite was a cafestol glucuronide conjugate. Recently, we studied the biotransformation of cafestol using a zebrafish (*Danio rerio*) water tank model.^15^ Five possible cafestol metabolites were suggested using liquid chromatography coupled to high-resolution mass spectrometry: 6-hydroxy-cafestol, 6,12-dihydroxy-cafestol, 2-oxo-cafestol, 6-oxo-cafestol, and one isomer whose position of the carbonyl group was not determined. Although these studies accounted for the hepatic metabolism of cafestol, none of them focused on the possible transformations that can occur in the gastrointestinal tract, as observed by De Roos et al.^11^ Most of the studies regarding cafestol metabolism employ a standard instead of the coffee brew due to the chemical complexity of the coffee brew.^13−15^

Oral bioavailability is essential to the activity of food-bioactive compounds. It is usually evaluated based on three main factors: bioaccessibility, absorption, and transformation of biomolecules.^16^ Bioaccessibility is the amount of ingested components available for absorption across the intestinal epithelia.^17^ This term encompasses the transformations undergone by the substance during digestion, absorption, and assimilation by the intestinal epithelium, as well as presystemic, intestinal, and hepatic metabolism.^18^ Bioaccessibility is typically assessed through *in vitro* digestion protocols that simulate digestion phases (oral, gastric, and intestinal duodenal). These protocols may or may not be followed by a study of absorption evaluation such as an assay using Caco-2 cells. Bioaccessibility assays are extremely relevant, as they investigate the interaction between nutrients and the food matrix while examining the effect of pH and digestive enzymes on potential nutrients.^19^ Moreover, the elucidation of cafestol degradation along the gastrointestinal tract and its digestion products is an important step in characterizing this diterpene metabolism, as these substances can reach the bloodstream, organs, and tissues after intestinal absorption and exert potential effects.

Thus, this study aimed to evaluate the bioaccessibility of cafestol from coffee brew and investigate its metabolism in an *in vitro* digestion model using liquid chromatography coupled to high-resolution mass spectrometry.

## 2 Materials and Method

### 2.1 Chemicals and Reagents

Acetonitrile, methanol, methyl-*tert*-butyl ether (LC-MS grade), and ethyl ether were obtained from BioGrade (Durham, USA). Formic acid and ammonium formate (LC-MS grade) were purchased from Tedia (Fairfield, USA). Ultrapure water with a resistivity of 18.2 M Ω/cm was obtained using a Millipore Milli-Q water purification system (Billerica, MA, USA). Hydrochloric acid (HCl), dimethyl sulfoxide (DMSO), sodium hydroxide (NaOH), potassium hydroxide (KOH), sodium chloride (NaCl), and sodium Bicarbonate (NaHCO_3_), as well as the enzymes and bile acid mixture from ovine and bovine, were purchased from Sigma-Aldrich (St. Louis, MO, USA). Raw coffee beans (*Coffee arabica* L.) from the 2018–2019 harvest year were obtained from a farm in the city of São José do Vale do Rio Preto, Brazil (22°11′35,2″ S, 42°59′08,6″ W).

Cafestol (>99% purity) was isolated from raw coffee beans (*Coffea arabica* L.) according to a protocol developed by our group.^20^ MS, ^1^H- and ^13^C NMR, and melting point data are shown in the Supporting Information.

### 2.2 Preparation of the Boiled Coffee Brew

The bioaccessibility studies were performed using boiled coffee since this type of brew has the highest amount of cafestol.^8,21,22^ The boiled coffee brew was prepared according to the International Agency for Research on Cancer.^23^ Thus, 100 g of green coffee beans were roasted using a CBR-101 roaster (Gene Café), following the manufacturer’s instructions for a light roast (12 min at 230 °C). Beans were weighed before and after roasting to assess the mass loss, and difference of up to 14% indicates a light roast.^24^ Subsequently, the beans were grounded using an analytical mill (IKA A11 basic), and particle size was determined using fixed-size sieves ranging from 850 to 1000 mm (Bertel). Seven grams of roasted and ground coffee were boiled with 100 mL of filtered water for 10 min, followed by a 2 min resting period, to allow the ground particles to settle at the bottom of the container. The prepared beverage was carefully transferred to a 100 mL amber glass bottle and frozen at −20 °C until analysis.

### 2.3 Quantification of Cafestol in the Boiled Coffee Brew

Cafestol was quantified in the boiled coffee brew according to the protocol described by Moeenfard et al.^9^ Initially, 2.5 mL of the beverage were saponified at high temperature with 3 g of potassium hydroxide in 4 mL Teflon-capped vials. The reaction was carried out at 80 °C for 60 min on a silica carbide plate with stirring on a heating plate. After the vials were cooled, four liquid–liquid extractions were performed using 2 mL of ethyl ether. The ether fraction was collected and washed with 5 mL of a 2 M sodium chloride solution. Subsequently, the solvent was evaporated under nitrogen flow, and the resulting solid was dissolved in methanol (4 mL). The sample was filtered through a 0.2 μm pore size PVDF filter and then subjected to analysis by high-resolution liquid chromatography coupled to a diode array detector (HPLC-DAD) (a Shimadzu 20A LC system equipped with a diode array detector SPD-M20A). The chromatographic conditions used were a reverse-phase column (Eclipse XDB C_18_ 150 × 4.6 mm; 5 μm) with acetonitrile and water (55:45) at 0.7 mL min^–1^ as the mobile phase. The sample injection volume was 20 μL, and the wavelength used was 220 nm.^25^ Cafestol was identified in the samples by comparing the retention time and UV spectra to those of the isolated cafestol standard. All analyses were performed in triplicate, and only results with a coefficient of variation equal to or less than 5% were accepted.

Quantification was performed using an external analytical curve of cafestol (10, 40, 70, 100, 130, 160, and 190 μg mL^–1^). The method was validated according to the FDA guidelines (Food and Drug Administration). The evaluated parameters were linearity, limits of detection (LOD) and quantification (LOQ), accuracy and precision, recovery, and selectivity.

### 2.4 *In Vitro* Digestion of the Boiled Coffee Brew

*In vitro* gastrointestinal digestion of the boiled coffee brew followed an adaptation of the harmonized protocol for digestion set out by INFOGEST.^19^ The activities of all of the enzymes used in the experiment were dosed according to the established protocols.

Two and a half milliliters of the beverage were mixed in a flask with 2.5 mL of fresh human saliva obtained from a volunteer for 2 min. The saliva donation followed the appropriate protocols to protect the rights and privacy of the donor, who consented to participate in the research. The saliva was obtained from a donor with a fast period of at least 2 h and without oral hygiene with toothpaste for at least the same amount of time. The collection was performed during the period necessary to collect the amount of saliva used in the study. No stimulation was used.

Subsequently, 2.5 mL of pepsin solution (423 U mg^–1^, Sigma-Aldrich, St. Louis, MO, USA) at a concentration of 2000 U mL^–1^ was added, and the pH was adjusted to 2 with a 0.1 M HCl solution. This solution was incubated in a Dubnoff bath (Thermo Fisher) at 37 °C for 120 min. After this gastric phase, 5 mL of a solution of porcine pancreatin (7.05 U mg^–1^, Sigma-Aldrich, St. Louis, MO,USA) and bile (1.00 mmol.g^–1^, Sigma-Aldrich, St. Louis, MO, USA) were added, and the pH was adjusted to 7 with a 0.1 M NaHCO_3_ solution. The solution was then incubated for 120 min at 37 °C. At the end of intestinal digestion, the samples were frozen at −20 °C until analysis. All analyses were performed in triplicate.

### 2.5 Determination of the Bioaccessibility of Cafestol after Digestion of the Boiled Coffee Brew

For the determination of cafestol bioaccessibility in boiled coffee brew after digestion, samples were centrifuged at 4500 rpm for 30 min and the supernatant was collected and considered the bioaccessible fraction of the intestinal phase. Subsequently, a liquid–liquid extraction of 2.5 mL from the intestinal phase was performed with 2 mL of ethyl ether. The solvent was evaporated under nitrogen flow, and the resulting solid was dissolved in methanol (1 mL). Chromatographic analysis followed the procedure established for cafestol analysis in the beverage (Section 2.3). Although only the intestinal phase was used to calculate the bioaccessibility fraction, the oral and gastric phase samples were also analyzed to monitor this molecule during the *in vitro* digestion process. All analyses were performed in triplicate, and only results with a coefficient of variation equal to or less than 5% were accepted.

### 2.6 *In Vitro* Digestion of Cafestol

For the analysis of the metabolites formed during the digestion of cafestol, an *in vitro* digestion protocol was performed using 1 mg of cafestol isolated from raw coffee beans previously dissolved in 100 μL of DMSO instead of the boiled coffee brew. The same procedures as those described in items 2.4 and 2.5 were used. The samples obtained from the oral, gastric, and intestinal phases were analyzed by liquid chromatography coupled with high-resolution mass spectrometry (LC-HRMS) to investigate cafestol metabolites (Section 2.7).

### 2.7 Liquid Chromatography–High-Resolution Mass Spectrometry (LC-HRMS) for Cafestol Metabolites

LC experiments were performed on Thermo Scientific Dionex Ultimate 3000 equipment (Thermo Fisher Scientific, USA) in a reversed-phase column (Syncronis C_18_ 50 mm × 2,1 mm × 1,7 μm). The mobile phase consisted of Milli-Q water with 0.1% formic acid and 5 mM ammonium formate (mobile phase A), and methanol containing 0.1% formic acid (mobile phase B) in a gradient of 15% B (0 min), 15–50% B (1–6 min), 50–95% B (6–9 min), 95% B (9–12 min), and 15% B (12.1–16 min). The flow was at 0.35 mL min^–1^, the injection volume was 5 μL, and the column oven temperature was 40 °C.

Mass spectrometry analysis was performed using a Q-Exactive Hybrid Quadrupole-Orbitrap mass spectrometer (Thermo Fisher Scientific, USA) equipped with an ESI source, spray voltage ±3.9 kV, ion transfer capillary 400 °C, sheath, and auxiliary gases at 50 and 15 arbitrary units.

The metabolites were investigated in positive ion mode using full scan (Full-MS) over the range of *m*/*z* 100–1000 at 70.000 resolution, followed by a ddMS2 Top 3 experiment at a resolution of 17.500. The collision energy (CE) ramp was (20–60 eV). In addition, Parallel Reaction Monitoring (PRM) of the protonated cafestol ion [M + H]^+^*m*/*z* 317.2111 was used to monitor the substance in the samples with a mass tolerance of 6 ppm.

The equipment was calibrated in positive mode with the manufacturer’s calibration solution (Thermo Fisher Scientific, Germany) of dodecyl sodium sulfate (*m*/*z* 265.1479), sodium taurocholate (*m*/*z* 514.2842), and ultra mark polymer 1621 (*m*/*z* 1279.9973, 1379.9905, 1479.9849, 1579.9778, 1679.9723, and 1779.9648).

### 2.8 Data Analyses

Data analyses were performed using Thermo Scientific Xcalibur 4.3 software (Thermo Fisher Scientific, USA).

A metabolite database was created using cafestol metabolites described in the literature^13−15^ and metabolites suggested by the *in silico* metabolism prediction software Way2Drug (created by multidisciplinary research from Way2Drug portal, 2010)^26^ and SMARTCyp 2.3 (Cambridge University, Cambridge, UK, 2013).^27^

## 3 Results and Discussion

### 3.1 Quantification of Cafestol in the Boiled Coffee Brew

Cafestol was quantified in the boiled coffee brew prior to its application in *in vitro* digestion assays to calculate its bioaccessibility. An external calibration curve was constructed using isolated cafestol in methanol at concentrations ranging from 10 to 190 μg mL^–1^ (*R* = 0.9965). Further information on the validation parameters is provided in the Supporting Information.

The extraction protocol employed involved a saponification step at the beginning of the procedure.^9^ This step is necessary because cafestol occurs in coffee brews mainly as esters of fatty acids (up to 95%, Figure 1A).^6^ Thus, saponification is used to convert the many cafestol esters into cafestol alcohol and simplify the analysis. This strategy has been widely used in the literature.^7,9,21,28−30^

Cafestol content was 130.06 ± 3.66 mg L^–1^ in a boiled coffee brew. In the literature, the cafestol content in boiled coffee brews varies widely from 5.2 to 128.8 mg L^–1^.^9,21,22,28,29,31^ This variability can be explained by the lack of standardization in brew preparation as well as different degrees of roasting, granulometry, boiling time, and a grain/water ratio used in the different studies.^32^ Furthermore, different aspects related to edaphoclimatic conditions and postharvest methods are also known to affect coffee diterpene content.^33^

### 3.2 Bioaccessibility of Cafestol after *In Vitro* Digestion of the Boiled Coffee Brew

To evaluate the bioaccessibility of cafestol, the *in vitro* digestion protocol was carried out using boiled coffee brew. The brew was used without the saponification protocol as we hypothesized that the lipases present in the intestinal digestion phase would hydrolyze the cafestol esters releasing free cafestol (alcohol form) into the intestinal fluid.

Lipases (E.C. 3.1.1.3) are acyl hydrolases present in most organisms such as microorganisms, plants, and animals. In the gastrointestinal tract, these enzymes are responsible for the hydrolysis of triglycerides (TAGs), diglycerides (DAGs), and monoglycerides (MAGs) into free fatty acids (FFAs), facilitating lipid absorption.^34^ However, these lipases are also capable of cleaving the ester bonds in other exogenous molecules.^35^

**Figure 2:**
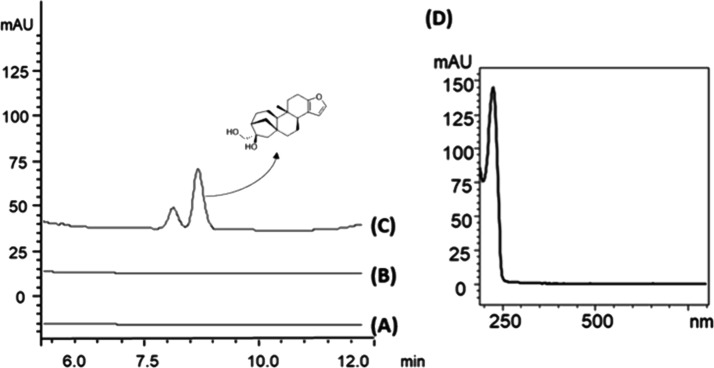
Chromatograms obtained by HPLC-DAD analysis of (A) oral, (B) gastric, and (C) intestinal phases of the *in vitro* digestion of boiled coffee brew; (D) UV spectra of the substance of a retention time of 8.68 min, corresponding to cafestol.

Thus, we evaluated the presence of cafestol in all phases of the *in vitro* digestion model. As shown in Figure 2, free cafestol was found only in the intestinal digestion phase (duodenal). This was accepted because the *in vitro* digestion protocol used in this study only contained lipase in the intestinal phase.^19^ Our results show that, although cafestol is found mainly in the form of esters in coffee brew, the lipases in the intestinal fluid can also cleave the ester bond, releasing free cafestol for absorption. Thus, the bioaccessible cafestol form is the cafestol alcohol. These results are interesting, as most studies investigating the biological activities of coffee diterpenes employ cafestol rather than cafestol esters.^3,10,36^ This is the first study to investigate the bioaccessibility of cafestol and indicates that this diterpene is absorbed mainly in its alcohol form.

In addition, the bioaccessibility of cafestol was calculated by comparing the amount of cafestol in the boiled coffee brew before digestion and in the intestinal phase after the digestion protocol was completed. The bioaccessibility of free cafestol in the boiled coffee brew was 93.65%.^18^

The bioaccessibility of a substance can be influenced by several factors, such as the chemical nature of the compound and the matrix in which it is present. The integrity of the matrix also interferes with the release of phytochemicals into the intestinal lumen because bioaccessibility depends on the release of compounds from the food matrix. Thus, food processing can also affect the bioaccessibility of many compounds.^42^

The bioaccessibility of cafestol has already been studied in spent coffee grounds through *in vitro* digestion and was described as 13.39%.^12^ The coffee bean, even when ground, is a much more complex chemical matrix than the brew.^43,44^ For example, green coffee beans have approximately 5 g 100 g^–1^ of insoluble fiber and 10–15 g 100 g^–1^ of proteins, whereas this type of fiber is not found in beverages and the amount of protein is significantly reduced.^45^

Some studies have indicated that the bioaccessibility of lipid compounds is reduced when the food matrix contains insoluble fibers, as these types of fibers interfere with the formation of micelles necessary for the solubilization of lipids in the intestinal lumen.^42,46^ Therefore, the greater bioaccessibility of cafestol from the coffee brew than from the spent grounds could be explained by the absence of these compounds in the brew.

Another study investigated the bioaccessibility of cafestol from boiled coffee brew in ileostomized volunteers.^11^ After the beverage was drank, the fluid from the ileostomy bags was collected for 14 h and saponified, and free cafestol was quantified by HPLC-DAD to give a bioaccessibility of 70%. Although this result is closer to that obtained in this study (93.65%), the study of digestion in human volunteers is complicated and costly and the results can vary widely from person to person. Furthermore, human volunteers should be subjected to severe ethical considerations.^47^ Thus, *in vitro* digestion protocols have been developed and are preferred for food, nutrition, and medical studies. *Invitro* models are quicker, cheaper, and have higher reproductivity than *in vivo* studies.^17,48^

### 3.3 Cafestol Metabolite Formed during *In Vitro* Digestion by LC-HRMS

The isolated cafestol was subjected to an *in vitro* digestion protocol to study the possible biotransformation of the cafestol alcohol during digestion.

**Figure 3:**
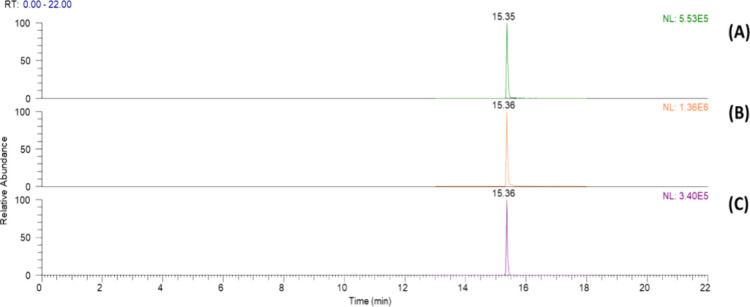
Base peak chromatogram for *m*/*z* 317.21112 [M + H]^+^ of LC-HRMS analysis of (A) oral, (B) gastric, and (C) intestinal phases of the *in vitro* digestion of cafestol.

The oral, gastric, and intestinal phases were analyzed using LC-HRMS. Cafestol was observed in all phases of digestion, represented by the ion at *m*/*z* 317.21112 [M + H]^+^ and a retention time of 15.36 min, as shown in Figure 3. Thus, cafestol is to a certain extent resistant to the digestive conditions to which it was subjected in this study.

The fragmentation spectrum of cafestol obtained by LC-HRMS was compared to the spectra obtained during the analysis of isolated cafestol and the spectra found in the literature.^15,49^ The fragmentation spectra and proposed fragmentation for cafestol are provided in the Supporting Information.

Initially, from the fragmentation spectrum of the hydrogen adduct, it was possible to observe the molecular ion at *m*/*z* 317.2104, referring to the protonated cafestol molecule. Next, we have fragments *m*/*z* 299.2005 and *m*/*z* 281.18942 referring to the loss of one and two water molecules, respectively, as reported for the cafestol molecule using atmospheric pressure chemical ionization (APCI). The fragment at *m*/*z* 253.15782 is formed by the opening of the B and C rings by the retro-Diels–Alder reaction and subsequent elimination of the side chain by remote hydrogen rearrangement. Furthermore, fragments with *m*/*z* values of 149.0959 and 147.08017 were described as marker fragments of the furan ring. The fragments *m*/*z* 131.08546 and *m*/*z* 121.10121 are derived from the breaking of the C-18 bond with oxygen, followed by dehydration of fragment *m*/*z* 149.09593 and breaking of the C-3 bond with oxygen, with the elimination of carbonyls from the same fragment, respectively. Finally, fragment *m*/*z* 81.0392, the base peak of the spectrum, corresponds to the portion of the furan ring linked to an ethylene group.

One cafestol metabolite, with *m*/*z* 331.19075 [M + H]^+^ and a retention time of 7.87 min, was observed during the *in vitro* digestion of cafestol. This metabolite was observed in the oral phase, and its intensity increased during the other phases. The extracted chromatogram is shown in the Supporting Information. The mass difference between cafestol and this metabolite may correspond to the addition of an oxo group (13.95926 Da).

**Figure 4:**
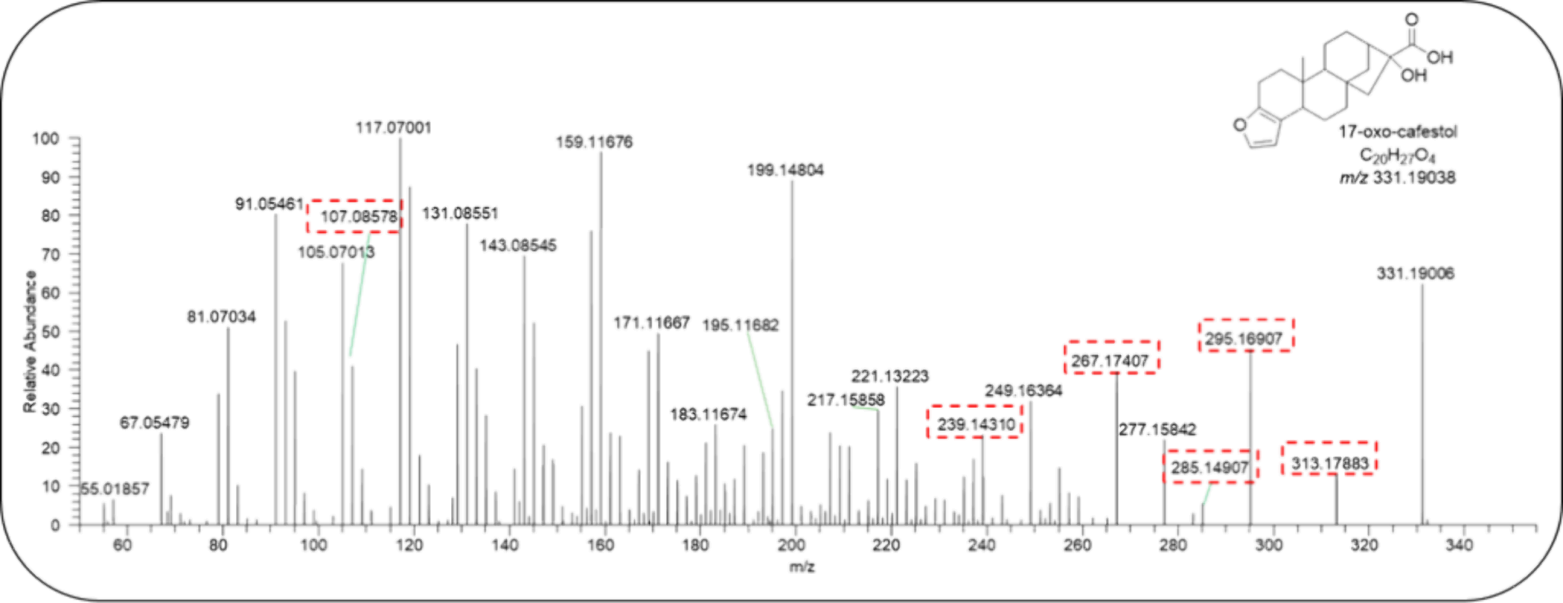
Structure and fragmentation profile of the cafestol metabolite (retention time 7.8 min) and *m*/*z* 331.19094 [M + H]^+^ by ESI-Orbitrap.

The possible structure of a carboxylic acid from cafestol (17-oxo-cafestol) and the mass spectrum obtained by the fragmentation experiment of this metabolite are presented in Figure 4.

**Figure 5:**
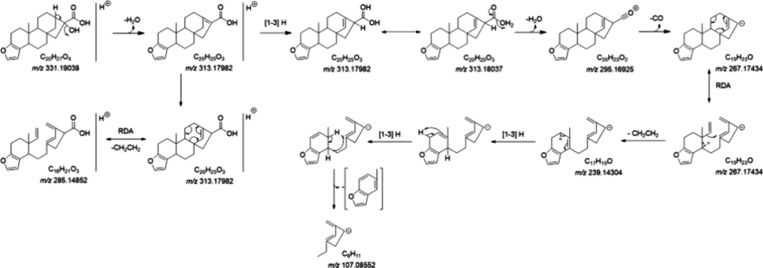
Proposed fragmentation of the carboxylic acid (17-oxo-cafestol) of cafestol by ESI-Orbitrap.

Fragments at *m*/*z* 313.17982 [M+H–H2O]^+^ and *m*/*z* 295.16925 [M+H-2H2O]^+^ correspond to two consecutive dehydrations from *m*/*z* 331.19038 [M + H]^+^. Fragment *m*/*z* 267.17434 [M+H-2H2O–CO]^+^ indicates the loss of a carbonyl group after dehydration. Furthermore, fragment *m*/*z* 239.14304 suggests that the carbonyl would be present in C-17 due to the loss of a CH_2_CH_2_ unit from ion *m*/*z* 267.17434, and ion *m*/*z* 285.14852 suggests a COOH in the metabolite structure. The MS fragmentation is rationalized in Figure 5. Oxidation at C-17 in cafestol is suggested by *in silico* investigations using SMARTCyp and Way2Drug programs for analyte target prediction, based on previous work from our group as detailed in Andriolo et al.

Isomers of 17-oxo-cafestol were described in the investigation of cafestol biotransformation in a zebrafish water tank (ZWT), determined by ultraperformance liquid chromatography coupled to high-resolution mass spectrometry with the support of an *in silico* approach.^15^ The absence of the ion at *m*/*z* 285.14852 in the mass spectra of the metabolite suggests that the oxo group is not positioned at the C-17 position. Thus, the authors suggested the metabolite 2-oxo-cafestol based on the rationalization of the mass spectrum.

Therefore, the carboxylic acid derivative (17-oxo-cafestol) from cafestol seems to be the most likely chemical structure for the metabolite according to the rationalization used in this study due to the presence of the ion *m*/*z* 285.14852 in the substance mass spectra. This study is the first to report this substance as a cafestol metabolite. The formation of 17-oxo-cafestol in the oral phase can be associated with the presence of microorganisms in saliva used in this study, which are derived from the oral microbiota.

The metabolite 17-oxo-cafestol is formed by the oxidation of the cafestol molecule in the oral phase of *in vitro* digestion. Saliva has many functions, such as lubrication, predigestion, protection, and taste modulation. Human saliva contains many reactive oxygen species (ROS) to control the oral microbiota.^50^ However, the production of salivary ROS is carefully managed by many enzymes to maintain the homeostasis of the oral cavity.^51^ Furthermore, saliva contains enzymes that can promote oxidation reactions, such as salivary peroxidase and superoxide dismutase.^52^ These enzymes are capable of oxidizing several xenobiotics and are one of the main lines of microbiological defense in the oral cavity.^53^ A study observed that several peroxidases were capable of promoting oxidative ring expansion of substituted furfuryl alcohols.^54^ Therefore, due to the oxidative capacity of this enzyme, it is possible that 17-oxo-cafestol was formed by the enzymatic oxidation of cafestol in the oral phase of the *in vitro* digestion protocol.

Moreover, many nutrients are involved in the regulation of the oxidative stress, such as vitamins C and E. Polyphenols present in tea and berries can raise the antioxidant capacity of saliva. Resveratrol, a polyphenol found in wine, is reported to also increase the antioxidant activity of the salivary fluid and protect salivary glands and salivary proteins from oxidative stress.^50^ As for coffee, caffeine impacts the oxidative homeostasis in saliva. Caffeine inhibits the antioxidant enzyme aldehyde dehydrogenase in saliva, leading to an increase in secretions of salivary aldehyde dehydrogenase and glutathione transferase.^55^

The bioaccessibility of cafestol from the boiled coffee brew and the metabolism of cafestol were studied by using an *in vitro* digestion method. During the digestion of the coffee brew, free cafestol was only observed in the intestinal phase with a bioaccessibility of 93.65%. However, other lipases, such as gastric lipases, not present in this study, could also cleave the ester bonds in humans. This suggests that cafestol is absorbed mainly in its free form (alcohol instead of ester, the main natural form found in coffee beans) due to the action of pancreatic lipase in the intestinal phase.

Furthermore, a novel cafestol metabolite formed during the digestion protocol was identified. The metabolite 17-oxo-cafestol (*m*/*z* 331.19038 [M + H]^+^) was formed during the oral phase of digestion probably due to the oxidation of the cafestol molecule. The structure of 17-oxo-cafestol was proposed through rationalization of the fragmentation mass spectra of the substance. To the best of our knowledge, this is the first study employing LC-HRMS to study the biotransformation of a substance during an *in vitro* digestion protocol. The investigation of these transformations is of great relevance, as they can affect the bioavailability of cafestol and its biological activities after absorption.

## Data Availability

Data will be made available on request.

## Supporting Information Available

Spectral information for the isolated cafestol standard, validation parameters for the chromatographic conditions employed, fragmentation spectra for cafestol and proposed fragmentation for the substance, table for the mass error (ppm) table for cafestol and 17-oxo cafestol fragments, extracted ion chromatogram for the metabolite 17-oxo-cafestol ([M + H]^+^= 331.19094) in oral, gastric, intestinal phases, structures for the cafestol metabolites investigated suggested by SMARTCyp e Way2Drug programs and the literature, and cafestol and kahweol chromatogram, and UV–vis spectra obtained by HPLC-DAD (PDF)

## Abbreviations

LC-MS: liquid chromatography coupled to mass spectrometry
DMSO: dimethyl sulfoxide
HPLC-DAD: high-performance liquid chromatography coupled to diode array detector
UV: ultraviolet
FDA: Food and Drug Administration
LOD: limit of detection
LOQ: limit of quantification
SGF: simulated gastric fluid
SIF: simulated intestinal fluid
LC-HRMS: liquid chromatography coupled to high-resolution mass spectrometry
PRM: Parallel Reaction Monitoring
TAGs: triglycerides
DAGs: diglycerides
MAGs: monoglycerides
FFAs: free fatty acids
EIC: extracted ion chromatogram
APCI: atmospheric pressure chemical ionization
ESI: electrospray ionization
ZWT: zebrafish water tank.
