# Supplementary material for: Bioaccessibility of Cafestol from Coffee Brew: A Metabolic Study Employing an In Vitro Digestion Model and LC-HRMS
Source: J Agric Food Chem. 2024 Dec 4;72(50):27876–83. doi: 10.1021/acs.jafc.4c06411 (PMC11660246; doi:10.1021/acs.jafc.4c06411)
Supplement: Supplementary file 1 — jf4c06411_si_001.pdf [file jf4c06411_si_001.pdf]

Supplementary Material

Bioaccessibility of cafestol from coffee brew: a metabolic study employing an *in vitro* digestion model and LC-HRMS

Ana Brand [a]; Ana Silva.[a]; Cyrus Andriolo[b.], Caroline Mellinger[c]; Thaís Uekane [d.], Rafael Garrett[a]; Claudia Rezende[a]*

[a] *Instituto de Química, Universidade Federal do Rio de Janeiro, Rio de Janeiro, Brasil - 21941-909*

[b] *L'Oréal Brazil, Rio de Janeiro, Brasil - 21044-020*

[c] *EMBRAPA Agroindustria de Alimentos, Rio de Janeiro, Brasil - 23020-470*

[d] *Departamento de Bromatologia, Escola de Farmácia, Universidade Federal Fluminense, Niterói, Rio de Janeiro, Brasil - 24241-002*

[*]Laboratorio de Análise de Aroma, Av. Athos da Silveira Ramos, 149, 626A - Cidade Universitária, Rio de Janeiro - RJ, 21941-909, Brasil.
(*claudia.rezendeufrj@gmail.com)

Cafestol (C20H28O3): white solid; m.p. 153.5–154.0 °C; 1H NMR (500 MHz, CDCl3, δ ppm): 7.25 (1H, d, J = 1.8 Hz, H19), 6.22 (1H, d, J = 1.8 Hz, H18), 5.31 (1H, s, C16–OH), 3.82 (1H, d, J = 11.1 Hz, H17a), 3.70 (1H, d, J = 11.1 Hz, H17b), 2.62 (2H, dd, J = 5.8, 2.7 Hz, H2), 2.27 (1H, dq, J = 12.6, 2.7 Hz, H5), 2.07–2.03 (3H, m, H13, H1, H14), 1.82 (1H, ddd, J = 12.9, 6.2, 3.1 Hz, 455 H6), 1.74–1.51 (10H, m, H6, H7, H11, H12, H14, H15), 1.27–1.23 (1H, m, H1), 1.19 (1H, d, J = 7.7 Hz, H9), 0.84 (3H, s, H20). 13C NMR (500 MHz, CDCl3, δ ppm): 148.8 (C, C3), 457140.7 (CH, C19), 120.2 (C, C4), 108.4 (CH, C18), 82.1 (C, C16), 66.4 (CH2, C17), 53.4458 (CH2, C15), 52.2 (CH, C9), 45.5 (CH, C13), 44.8 (C, C8), 44.3 (CH, C5), 41.0 (CH2, C7), 38.7 (C, C10), 38.3 (CH2, C14), 35.8 (CH2, C1), 26.2 (CH2, C12), 23.2 (CH2, C6), 20.8 (CH2, C2), 19.1 (CH2, C11), 13.5 (CH3, C20). HRMS (ESI+) for protonated cafestol [M+H] + = C20H29O3. Exact Mass: 317.2111, found 317.21057 (error 1.67 ppm). Spectral information for the cafestol standard. All the results are in accordance with the literature (Novaes et al., 2020; Lima et al., 2020).

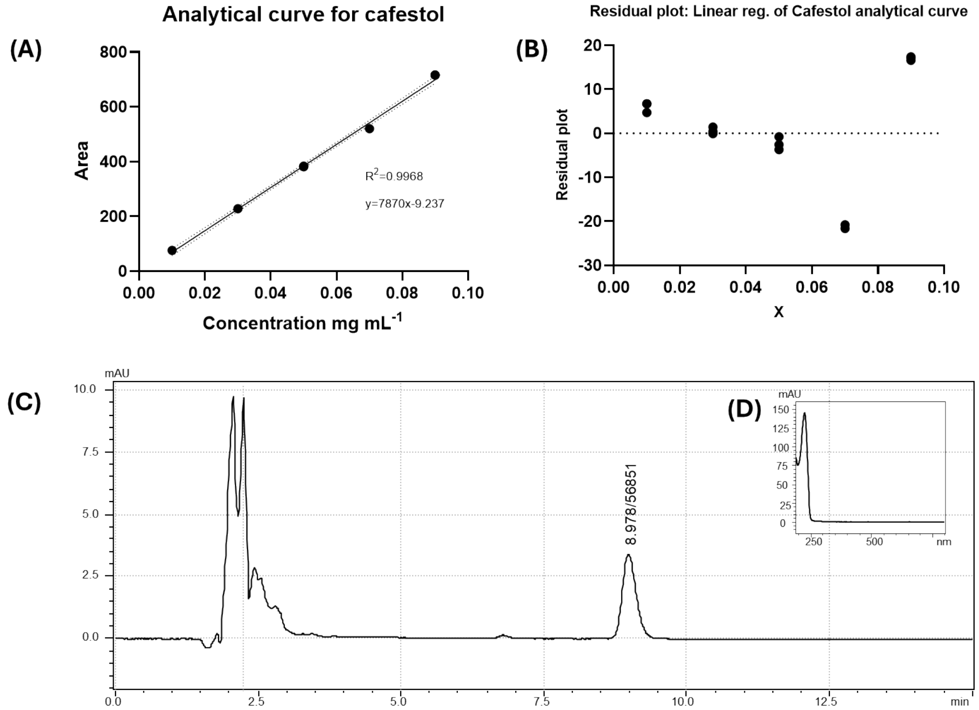

Figure S1: (A) Analytical curve for cafestol; (B) Residual plot for the analytical curve of cafestol; (C) Chromatogram obtain for the analysis of a cafestol standard by HPLC-DAD at 220 nm; (D) UV-Vis spectrum for the cafestol standard.

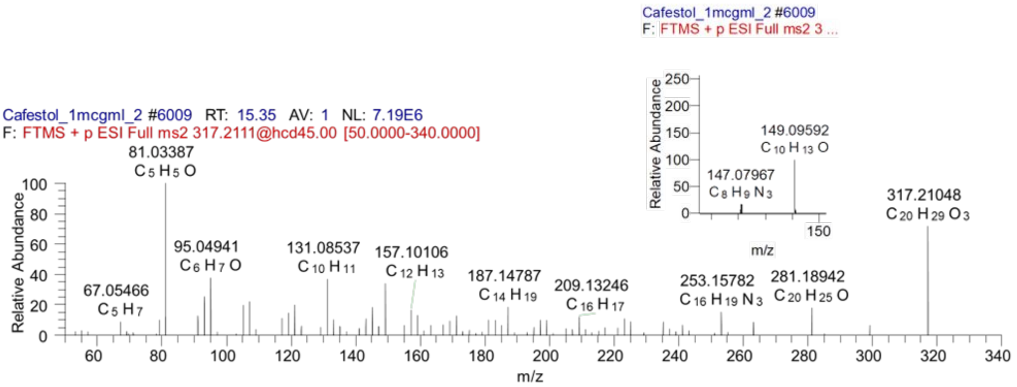

Figure S2: Fragmentation spectrum for cafestol obtained by LC-HRMS.

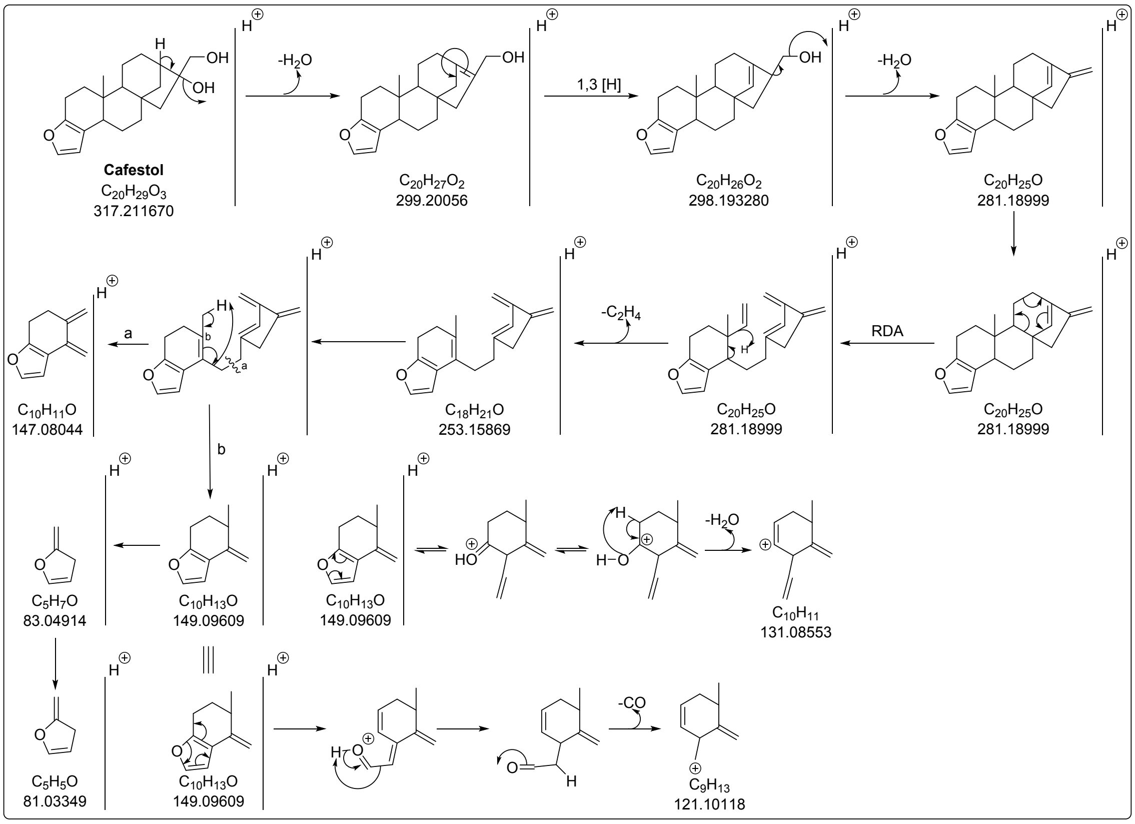

Figure S3: Proposal for the fragmentation of cafestol by LC-Orbitrap according to Andriolo et al., 2021.

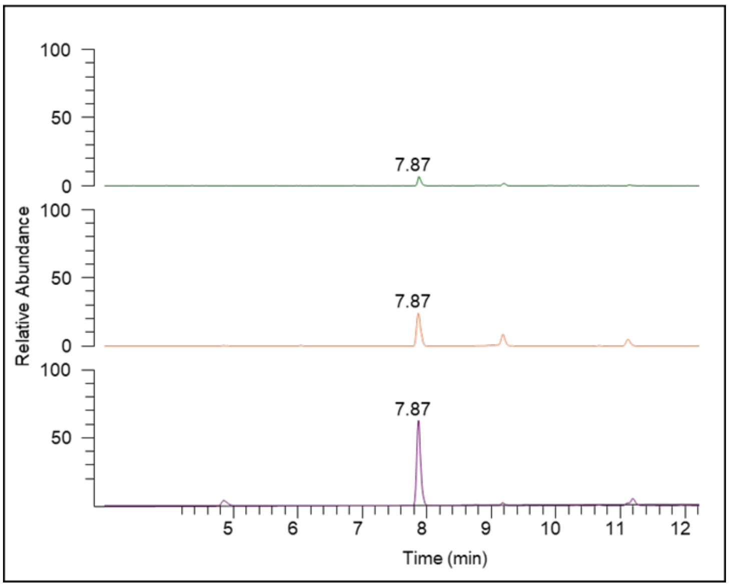

Figure S4: Extracted ion chromatogram (EIC) for the cafestol metabolite [M+H]+ = 331.19094 in the analysis of the digestion phases.

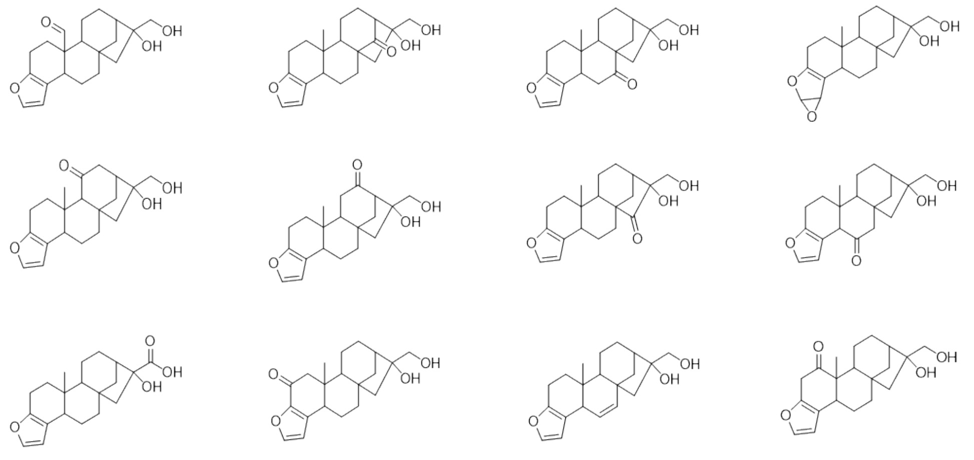

Figure S5: Cafestol metabolites suggested by the programs Way2Drug, SMATCyp, Van Crutchen et al., 2010; and Andriolo et al., 2021).

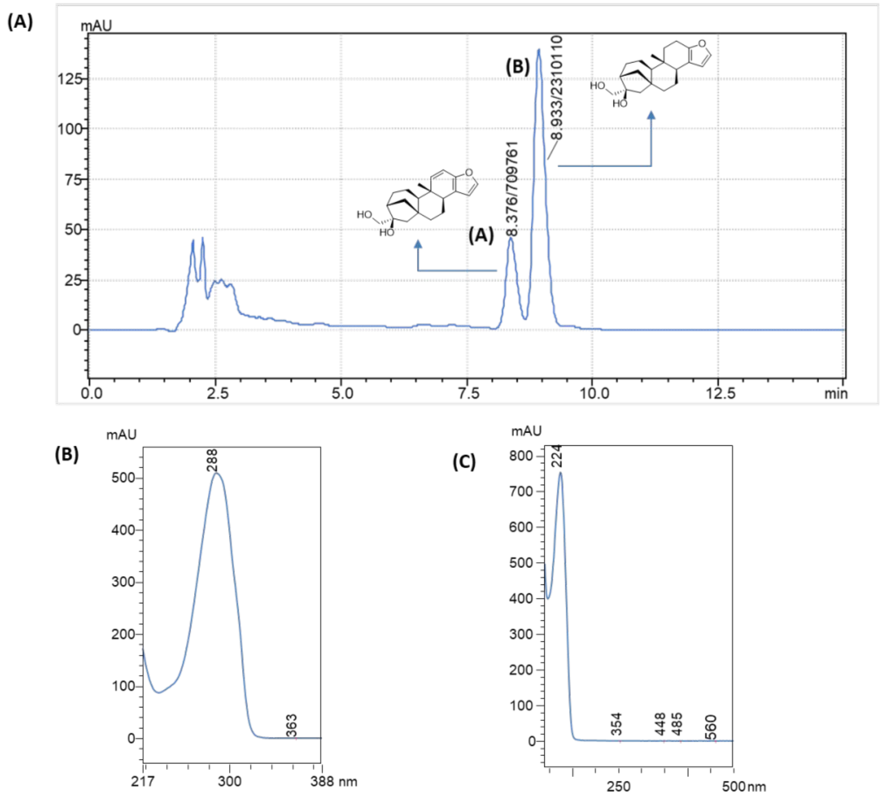

Figure S6: (A) Chromatogram of the boiled coffee brew obtained by high-resolution liquid chromatography coupled to a diode array detector (HPLC-DAD). The chromatographic conditions used were a reverse-phase column (Eclipse XDB C18 150 x 4.6 mm; 5 µm) with acetonitrile and water (55:45) at 0.7 mL min-1 as the mobile phase. The sample injection volume was 20 µL, and the wavelength used was 220 nm. **(B)** UV spectra of the substance of retention time of 8.37 minutes, corresponding to kahweol. **(C)** UV spectra of the substance of retention time of 8.93 minutes, corresponding to cafestol.

Table S1: Validation parameters for the quantification of cafestol in boiled coffee brews by HPLC-DAD

Parameter	Result
Linear range (mg mL^{-1})	0.01; 0.04; 0.07; 0.10; 0.13; 0.16; 0.19
Determination coeficient	0.9968
Correlation coeficient	0.9984
Cochcran´s test	Homoscedastic
Matrix effect	Absent
Limito of detection (LOD) (µg mL^{-1})	0.20
Limit of quantification (LOQ) (µg mL^{-1})	0.75
Recovery	90.78%
Intraday precision	95.37%

Table S2: Mass erros in ppm for cafestol and 17-oxo cafestol adducts and MS2 fragments.

**Cafestol**			
**Molecular Formula**	**Theoretical *m/z***	**Experimental *m/z***	**Mass error (ppm)**
$C_{20}H_{29}O_3$	317.21167	317.21048	3.751438
$C_{20}H_{27}O_2$	299.20056	299.201105	1.821521
$C_{20}H_{25}O$	281.18999	281.18942	2.027099
$C_{18}H_{21}O$	253.15869	253.15924	2.172550
$C_{10}H_{11}O$	147.08044	147.07967	5.235230
$C_{10}H_{13}O$	149.09609	149.09592	1.140204
C_5H_5O	81.03349	81.03387	4.689419
**17-oxo-cafestol**			
**Molecular Formula**	**Theoretical *m/z***	**Experimental *m/z***	**Mass error (ppm)**
$C_{20}H_{27}O_4$	331.19039	331.19006	0.996406
$C_{20}H_{25}O_3$	313.17982	313.17883	3.161123
$C_{20}H_{23}O_2$	295.16926	295.16907	0.643698
$C_{19}H_{23}O$	267.17434	267.17407	0.967138
$C_{17}H_{19}O$	239.14304	239.14310	0.250896
C_8H_{11}	107.08553	107.08578	2.334582
$C_{18}H_{21}O_3$	285.14852	285.14907	1.928819
